# Self and Other Mentalizing Polarities and Dimensions of Mental Health: Association With Types of Symptoms, Functioning and Well-Being

**DOI:** 10.3389/fpsyg.2021.566254

**Published:** 2021-02-03

**Authors:** Sergi Ballespí, Jaume Vives, Carla Sharp, Lorena Chanes, Neus Barrantes-Vidal

**Affiliations:** ^1^Department of Clinical and Health Psychology, Universitat Autònoma de Barcelona, Barcelona, Spain; ^2^Department of Psychobiology and Methodology of Health Sciences, Universitat Autònoma de Barcelona, Barcelona, Spain; ^3^Department of Psychology, University of Houston, Houston, TX, United States; ^4^Serra Húnter Programme, Generalitat de Catalunya, Barcelona, Spain; ^5^Department of Mental Health, Fundació Sanitària Sant Pere Claver, Barcelona, Spain; ^6^Centre for Biomedical Research Network on Mental Health, Instituto de Salud Carlos III, Madrid, Spain

**Keywords:** self-other mentalizing, polarities, symptoms, functioning, well-being, adolescents, non-clinical population, mental health

## Abstract

Research suggests that the ability to understand one’s own and others’ minds, or mentalizing, is a key factor for mental health. Most studies have focused the attention on the association between global measures of mentalizing and specific disorders. In contrast, very few studies have analyzed the association between specific mentalizing polarities and global measures of mental health. This study aimed to evaluate whether self and other polarities of mentalizing are associated with a multidimensional notion of mental health, which considers symptoms, functioning, and well-being. Additionally, the level or depth of mentalizing within each polarity was also analyzed. A sample of 214 adolescents (12–18 years old, *M* = 14.7, and SD = 1.7; 53.3% female) was evaluated on measures of self- (Trait Meta-Mood Scale or TMMS-24) and other- mentalizing (Adolescent Mentalizing Interview or AMI), multi-informed measures of psychopathology and functioning based on Achenbach’s system, and measures of psychological well-being (self-esteem, happiness, and motivation to life goals). Results revealed no association between mentalizing polarities and higher-order symptom factors (internalizing, externalizing, and global symptoms or “*p*” factor). Self-mentalizing was associated with self-esteem (*B* = 0.076, *p* < 0.0005) and motivation to life goals (*B* = 0.209, *p* = 0.002), and other-mentalizing was associated to general, social and role functioning (*B* = 0.475, *p* < 0.0005; *B* = 0.380, *p* = 0.005; and *B* = 0.364, *p* = 0.004). This association between aspects of self-other mentalizing and self-other function has important implications for treatment and prevention. Deeper mentalizing within each polarity (i.e., comprehension beyond simple attention to one’s own mental states, and mentalizing referred to attachment figures vs. mentalizing referred to the characters of a story) revealed stronger associations with functioning and well-being. Because mentalizing polarities are associated with functioning and well-being but not with symptoms, a new hypothesis is developed: mentalizing does not contribute to resiliency by preventing symptoms, but by helping to deal with them, thus improving functioning and well-being independently of psychopathology. These findings support that promoting mentalizing across development may improve mental health, even in non-clinical population.

## Introduction

One pivotal challenge of neuroscience is to understand how one mind knows other minds (Amodio and Frith, 2006; Carruthers, 2019). Consciousness about one’s own mental states (emotions, intentions, desires, and thoughts) and those of others is a high-order cognition that has been approached from several perspectives. Social intelligence (Thorndike and Stein, 1937), social cognition (Heider, 1967), Theory of Mind (Premack and Woodruff, 1978), mind-blindness (Baron-Cohen, 1990), Intra- and Inter- personal intelligence (Gardner, 1987), and Emotional Intelligence (Salovey and Mayer, 1993) are some of the myriad terms used across the Twentieth century to refer the human capacity to be aware of the states of the mind, i.e., the intentional states (emotions, feelings, and drives) that underpin human behavior (Allen et al., 2008; Choi-Kain et al., 2008). The proliferation of theories to approach this higher order cognition reveals a persistent interest in this capacity, but also contributes to a proliferation of cousin-concepts referred to the same brain function.

### Mentalization as a Multi-Dimensional Higher Order Cognition

In this context, the mentalization paradigm neither provides a new concept nor discovers a novel ability (Bateman and Fonagy, 2019), but organizes the field from a multidimensional perspective based on advances in neuroscience (Kim, 2015). Mentalization refers to the brain’s ability to keep the mind in mind (Frith and Frith, 2003). In other words, it defines our capacity to be aware of the intentional mental states such as emotions, feelings, intentions, and thoughts that underpin one’s own and others’ behavior (Frith and Frith, 2006). The mentalization paradigm provides a unified, transtheoretical and transdiagnostic perspective, and encompassing cousin-concepts organized around four polarities (Allen et al., 2008). According to this organization, mentalization can be automatic or controlled, cognitive or affective, internally or externally focused, and referred to one’s own (self) or others’ mental states (Luyten et al., 2020).

### Mentalization and Mental Health

The ability to consider mental states makes possible to understand human’s behavior and to navigate our complex social world (Freeman, 2016). By contrast, problems with this ability are associated with difficulties in social and psychological functioning (e.g., autism spectrum disorders) and poor of mental health (Katznelson, 2014). Further, research shows that mentalizing is non-specifically affected in the presence of psychopathology regardless the type of disorder (Sharp et al., 2008). Mentalizing problems are prominent in personality disorders, especially in borderline and antisocial personality disorder, as well as in depression, anxiety, social anxiety, trauma-related disorders, attachment disorders, eating disorders, addictions, somatoform disorders, autism, and psychosis (see Ballespí et al., 2018; Bateman and Fonagy, 2019; Luyten et al., 2020, for a review). This non-specific association suggests that mentalizing is decreased when mental health decreases. Possibly for this reason, mentalization is commonly targeted to restore mental health, making it a common factor in most psychological treatments (Allen et al., 2008; Fernández-Sotos et al., 2019). Taken together, this evidence suggests that mentalization is a key factor for mental health.

### The Current Study

To date, most studies have focused attention on the association between global measures of mentalizing and specific disorders. In contrast, very few studies have analyzed the association between specific mentalizing polarities and global measures of mental health. Moreover, the role of each polarity of mentalization is typically not evaluated. Measures of self- and other mentalizing are usually used interchangeably in the literature, and the level or depth of mentalizing is not often distinguished when comparing studies. To better understand the role of mentalizing in salutogenesis, the multidimensional nature of this capacity needs to be considered (Luyten et al., 2020).

The first aim of this study was to evaluate whether different polarities of mentalizing, specifically self- vs. other-mentalizing, are associated with different dimensions of mental health. Recent debates further developed the classic definition of World Health Organization, whereby mental health is not only the absence of mental disorders but also considers social- and role- functioning along with psychological well-being (World Health Organization (WHO), 2018). In fact, this wide-reaching definition of mental health has been refined considering *“*…*a dynamic state of internal equilibrium”* as well as the *“*…*ability to cope with adverse life events and function in social roles”* (Galderisi et al., 2015, 2017). Consistently, mental health was operationalized in the current study using the number of symptoms, one’s level of social- and role- functioning, and measures of psychological well-being (i.e., happiness, self-esteem, and transcendence or motivation to life goals). Basing on this multi-dimensional view, we hypothesized that self- and other polarities of mentalizing might be differently associated to self- and other- function.

### The Hypothetical Association Between Self- Mentalizing, Inner Functioning and Internalizing Problems

The capacity to be aware of one’s own mental states is useful to deal with one’s own suffering. Self-awareness or insight into one’s own mental states is a common ingredient in all psychological treatments (Allen et al., 2008). Specifically, self-mentalizing is important to be aware of- and to understand what one is feeling (Oldershaw et al., 2010; Lumley et al., 2017), to determine whether one’s emotional suffering decreases after an intervention (Davis et al., 2019), or to understand why we react the way we do toward others (Fonagy and Target, 1997; Frith, 1999; Brackett et al., 2006). Self-mentalizing also promotes self-regulation (Fonagy et al., 2005; Heatherton, 2011), thus suggesting a role in the “digestion” of one’s own feelings.

Because internalizing problems are defined as those in which suffering is directed inward (e.g., anxiety, depression, and somatic complaints; Achenbach et al., 2016), we hypothesized that “self” polarity of mentalizing would be especially associated with internalizing problems. Likewise, if self-mentalizing helps to manage one’s own mental states, and psychological well-being or internal equilibrium are usually associated with mental processes, we also predicted that self-mentalizing would be more strongly associated with measures of psychological well-being (i.e., happiness, self-esteem, and life-motivation) than would other-mentalizing.

### The Hypothetical Association Between Other-Mentalizing, Social-Functioning, and Externalizing Problems

The capacity to understand others’ mental states is an advantage to navigate in the social world (Amodio and Frith, 2006). An adequate interpretation of others’ minds is associated with better social and role functioning (Brackett et al., 2006; Perera and DiGiacomo, 2013; Miao et al., 2017), higher quality of social interaction (Lopes et al., 2004), and sociometric status (Slaughter et al., 2015; Lonigro et al., 2018), less inter-personal conflicts (García-Sancho et al., 2014), more secure attachment style (Fonagy and Bateman, 2016), and higher social support (Di Fabio, 2015). Because our social and role functioning occur in the social world, it was hypothesized that mentalization regarding others’ mental states might be more associated with social and role functioning than self-mentalizing.

Psychopathological problems expressed outside the individual and with a special impact in the social world are defined as externalizing problems (Achenbach et al., 2016) and involve symptoms with an particular impact on others (e.g., impulsivity, aggression, oppositionism, and rule-breaking behavior; Liu, 2004). Studies of Borderline Personality Disorder (Fonagy and Luyten, 2009; Sharp et al., 2011) and aggression (Dolan and Fullam, 2004; Taubner et al., 2013) show that mentalizing about others’ intentions and feelings is associated with externalizing symptoms. However, acting out is also associated with emotional dysregulation, which is in turn associated with problems of self-mentalizing (Fonagy et al., 2005). Thus, we hypothesized that both self- and other- polarities of mentalizing would be associated to externalizing symptoms.

### The Hypothetical Association Between Levels of Self- and Other- Mentalizing Polarities and Level of Mental Health

The degree or depth of mentalizing processes is not typically evaluated in research. The awareness of one’s own mental states can be operationalized either as simple attention to one’s own emotions (that is, the degree to which individuals notice their feelings) or as further comprehension or emotional clarity (that is, the ability to distinguish and to understand one’s mood; Mayer and Gaschke, 1988; Salovey et al., 1995). These levels of emotional awareness (e.g., attention vs. further comprehension) are usually assessed in separate studies or, more rarely, they are assessed in the same study but refer to a very specific psychopathological condition (e.g., Butler et al., 2018; Ballespí et al., 2019). However, no study has considered these different levels of emotional awareness in the context of general mental health, especially in a sample of non-clinical general population.

Similarly, mentalization about others’ mental states can be evaluated on a spectrum of profundity. Other-mentalization is measured using myriad methods that are often compared in research, in spite of the important differences existing among them (Luyten et al., 2019). Two primary groups of methods exist: Clinical interviews based on primary attachment figures, and film- or picture-based measures which are non-specific to the individual being evaluated. When using an attachment interview, mentalizing is evaluated with regard to well-known close-others such as family members and based on internal cues, which requires the individual to draw on complex representations of attachment figures (i.e., the working model; Fonagy and Target, 1997). In contrast, film- or picture-based measures utilize characters and stories to prompt reasoning for the motivations behind characters’ actions. In such a measure, there is not a complex representation of a specific well-known other, but mentalizing is based on general knowledge about social behavior and on the external cues of these characters like facial expression, gestures, and tone of voice (Frith and Frith, 2012).

It can be argued that mentalizing activity which refers to an “insignificant” character of a picture-based story is disparate to mentalizing activity of a real, close other. While a story character activates general social knowledge, a specific person, whom an individual holds an affective bond to (attachment relationship) activates a unique “working model” of the figure, with very concrete, complete and refined knowledge about the personality and social functioning of this specific close other (Collins et al., 2006). These different levels of mentalizing referred to others’ mental states, based on a different degree of specificity or depth, should be also distinguished. This distinction, however, is lacking from the literature, whereby different studies with inconsistent measures of mentalizing are compared.

Consequently, the second aim of this study was to analyze whether the level of self- or other- mentalizing is associated with symptoms, functioning and well-being. Regarding the self- polarity, attention vs. further comprehension of one’s own emotional states is distinguished in the relationship with symptoms, functioning and well-being. Regarding the other- polarity, mentalizing referred to the characters of a picture-based story (i.e., unspecific others) is distinguished from mentalizing referred to specific and real attachment figures, which is expected to be more refined (Fonagy and Target, 1997; Fonagy and Bateman, 2016).

We hypothesized that deeper levels of mentalizing (that is, a higher relative ability to mentalize) would be more strongly associated to mental health. Specifically, it was expected that comprehension of one’s own emotional states would be more strongly associated to internalizing symptoms and psychological well-being than simple attention (i.e., a less profound understanding of one’s own internal mental states). Further, mentalizing about attachment figures is more specific and refined, and it better reflects the degree of sophistication that someone is able to reach when mentalizing. Therefore, we predicted that attachment-based mentalizing, that is, mentalizing referred to significant close others (attachment figures) might be more strongly associated with externalizing symptoms and social- and role- functioning than mentalizing referred to the characters of a story (i.e., general or ‘insignificant others).

## Materials and Methods

### Participants

This study was based on a sample of adolescents from the general population. Importantly, mentalization is quite developed at adolescence. Furthermore, both psychopathological and a wide range of subclinical problems are quite prevalent in community adolescent samples (Merikangas et al., 2010; Muris et al., 2011).

Participants were recruited from schools within the context of a broader project about psychopathology, personality, and coping skills. The inclusion criteria were: (1) to be in the eligible age range of the study (12–18); (2) to speak Spanish or Catalan (i.e., the languages of the questionnaires). The exclusion criteria were: (1) presence of severe mental illness such as autism spectrum disorder, psychosis or intellectual disability; (2) parents, teachers or adolescents failing to fill in one or more scales. Five out of 10 invited schools agreed to participate, thus providing an eligible sample of 1735 families. Primary participation refusal reasons included low interest in the project, being too busy, or refusal to provide data about family’s mental health. The final sample consisted of 214 adolescents (53.3% of girls) aged 12 to 18 years old (*M* = 14.7, SD = 1.7). Approximately 87% of the participants were Caucasian (White-European), 9% Arabic, 2% Asian, and 2% Latino. Most adolescents came from families with middle socio-economic level (72%), although there was a bias to medium-high and high socio-economic level (10.3% Low, 13.6% Medium-Low, 18.7% Medium, 39.7% Medium-High, and 17.8% High) according to the Hollingshead’s index (Hollingshead, 1975).

### Measures

#### Mentalization

##### Adolescent Mentalizing Interview

This is a brief, two-part, semi-structured interview (Ballespí and Pérez-Domingo, 2015) specifically designed for adolescents and originally created in Spanish and Catalan to obtain an interview-based score of mentalization in a short time (i.e., 20–30 min depending on the responses of the participant). It consists of two guided exercises with 7 questions scored from 0 to 4, for which 0 indicates that mentalization is “*Absent”* in the individual’s response, 1 that it is “*Poor*,” 2 “*Sufficient,”* 3 “*Good,”* and 4 “*Sophisticated*.” The first part (3 items) is a story-based procedure used to ask the adolescent about the mental states of the characters of a fictitious illustrated story. In the second part, individuals are encouraged to choose two very-close-others (i.e., two people important for them at this moment of their life; Bartholomew and Horowitz, 1991) and different *demand* questions (Fonagy et al., 1998) are made in order to explicitly elicit mentalizing ability (e.g., *“Why do you think he reacted like that?”*). The first part is aimed to provide a measure of non-attachment-based mentalizing since it is referred to fictious unknown characters of a story (i.e., “insignificant” others). The second part is aimed to measure mentalization toward an attachment figure, i.e., a significant close other. Reliability in the present sample is good both for the total scale (α = 0.91) and the subscales (α = 0.88 and α = 0.85), respectively.

##### Trait Meta-Mood Scale

The Trait Meta-Mood Scale (TMMS-24) measures the 3 dimensions of meta-cognition defined through factor analysis according to the model of Mayer and Salovey (Mayer and Gaschke, 1988): attention, comprehension (emotional clarity), and repair (emotional regulation) toward one’s own emotional states. The widely used 24-items version was selected (Fernandez-Berrocal et al., 2004). All 24 items are scored from 1 (strongly disagree) to 5 (strongly agree). The first two dimensions of attention and emotional clarity or comprehension were used in the present study. The attention scale includes items such as *“I pay a lot of attention to how I feel*,” while the clarity scale evaluates the understanding of one’s emotional states and includes items like *“I almost always know exactly how I am feeling*,” or *“I can’t make sense out of my feelings*.” The internal consistency in the current sample is excellent (α = 0.91 for the total score, α = 0.90 for attention, and α = 0.92 for comprehension).

#### Psychopathology

##### Achenbach’s System for Empirically Based Assessment

Achenbach’s System for Empirically Based Assessment (ASEBA) is a well-known dimensional and empirically derived system to classify psychopathology with good psychometric properties (Achenbach, 2020a). Second order scales of general, internalizing and externalizing problems reported from parents’ and teachers’ were used in the current study (Achenbach, 2020b). The Spanish adaptation (Achenbach and Rescorla, 2001) shows excellent internal consistency both for the Child Behavior Check-List or CBCL (α ranges from 0.78 to 0.97) and the Teacher’s Report Form or TRF (α ranges from 0.72 to 0.97), as well as adequate test-retest reliability in both cases (ICC from 0.85 to 0.90 for CBCL, and 0.60–0.90 for the TRF).

Drawing from the complementary perspective provided by parents and teachers (De Los Reyes and Kazdin, 2005), multi-informant indices of symptoms and functioning were developed. Using principal component analysis, the standardized object scores in the one-factor solution were used to combine parents’ and teachers’ scores in one single measure. Thus, multi-informed indexes of *Total* (70% of explained variance; weights = 0.84; *α* = 0.58), *Internalizing* (68% of explained variance; weights = 0.82; *α* = 0.53), and *Externalizing problems* (67% of explained variance; weights = 0.82; *α* = 0.50) were obtained. The psychometric properties of these indices are adequate since they combine information from complementary (i.e., usually discrepant) informants (De Los Reyes et al., 2015).

#### Psychological Functioning

The CBCL/6-18 (parents’ form) provides 3 indices of functioning (Activities, Social, and School) along with one summarizing all of them (the total competence scale). The TRF/6-18 (teacher’s form) provides a scale of academic performance, four dimensions of adaptive functioning (Working Hard, Behaving, Learning, and Happy) and a total score based on those four (total adaptive functioning; see the profiles in Achenbach, 2020b). Using the same procedure as in the case of psychopathology described above, three multi-informed general indices of functioning were developed combining parents’ and teachers’ reports: *Social Functioning* (i.e., peer relationships, sociometric status, and involvement with family members; 55% of explained variance, weights from 0.62 to 0.81, *α* = 0.49), *Role Functioning* (i.e., performance in academic and extra-academic activities like sports or chores; 56% of explained variance, weights from 0.24 to 0.91, *α* = 0.73), and *General Functioning* (64% of explained variance, weights = 0.80, *α* = 0.45).

#### Well-Being

Modern conceptions of mental health agree on that it consists not only the absence of symptoms or disorders but a general state of social and emotional well-being (Bertolote, 2008; World Health Organization (WHO), 2018) or, in the more refined terms of Galderisi et al. (2017), *“*…*a dynamic state of internal equilibrium which enables individuals to use their abilities in harmony with universal values of society.”* According to these modern notions, three construct measures that are usually low in the absence of good mental health were evaluated: happiness (e.g., Ho et al., 2018), self-esteem (Pyszczynski et al., 2004; Keane and Loades, 2017), and transcendence (Nygren et al., 2005). As emotional well-being and self-esteem, motivation is decreased in most psychopathological conditions (e.g., Vogel et al., 2019), while positive motivational states (e.g., to have aspirations or goals in life) denote healthy personality functioning (Seligman and Peterson, 2006; Zhang and Yu, 2014). Therefore, we included a motivational measure based on the Aspiration index of Kasser and Ryan (2001), called here “transcendence,” and described below.

*Happiness* was measured through Teachers’ Report Form (Achenbach, 2020a) which includes a scale of happiness based on teachers’ ratings to the question “*How happy is he/she*?” using a 7-point scale (from *1-Much less* to *7-Much more*).

*Self-Esteem* was assessed with the widely used *Rosenberg’s Self-Esteem Scale* (RSES; Rosenberg, 1965), consisting of 10 items rated with 5-point scales according to the degree of agreement with each statement. The Spanish adaptation shows adequate psychometric properties (Martín-Albo et al., 2007). The internal consistency in the current sample was excellent (*α* = 0.90).

*Transcendence* is a scale based on the dimensions of Spirituality, Community, and Conformity of the Aspiration Index of Kasser and Ryan (2001; see also Grouzet et al., 2005) consisting of 12 items rated from 1 to 9 based on the importance and likelihood of different goals in the life of the participant. The Spanish adaptation show adequate psychometric properties (Romero et al., 2012). The internal consistency in the present sample is good (*α* = 0.79).

### Procedure

The study met ethical standards according to Declaration of Helsinki and the revision of the Ethics Committee of the Universitat Autònoma de Barcelona (CEEAH; Spain) (Ethical clearance number: CEEAH 2603). Accordingly, we reported how we determined and obtained our sample, all data exclusions, all manipulations, and all measures in the study. Families were informed about the objectives, relevance, and implications of the research by means of a letter circulated by the school. Parents, teachers and children were also invited to a meeting to resolve any questions or concerns regarding the study. All participants (i.e., parents, adolescents, and teachers) provided written informed consent and were offered the option of being informed in the case of one or multiple scales reflecting dysfunction of clinical significance. Data were recruited in the schools to simplify logistics. The participants (adolescents, parents, and teachers) received the questionnaires in closed envelops with their identities encrypted using alphanumeric codes and were given a deadline to return them. The instructions highlighted the importance and reasons that each informant should complete his or her form in in private, and of completing the task over the course of multiple sessions to buffer the effect of fatigue. All forms were reviewed and returned to the informants in case it was necessary to correct missing or out-or-range values. The interviews were carried out individually in private spaces provided by each school (e.g., an office, and empty classroom). Data collection occurred over the course of approximately 5 weeks in all schools included in the study. Paper responses were archived in the lab with the same alphanumeric codes they were entered to the data matrix.

### Statistical Analysis

We conducted power analyses using Stata V. 16.1. With α = 0.05, power (1–β) = 0.8, four explanatory variables, and three control variables, the sample needed to detect a minimum change of 0.05 in *R*^2^ was 208. All of the analyses were performed with sample size of 214. Linear regressions were conducted using IBM SPSS Statistics v24.0 package. The results of the association between mentalizing and each measure of symptoms, functioning and well-being are presented as linear regression coefficients (B) for quantitative responses, reporting 95% confidence intervals (95% CI), and *P*-values (*p*).

Because sex, age, and socio-economic level are associated with both mentalization and psychopathological conditions, these variables were controlled for in all analyses. Sex is closely related both to psychopathology (Zahn-Waxler et al., 2008) and mentalizing (Schulte-Rüther et al., 2008; Cheng et al., 2009). Mentalization is a high-order cognition that becomes more complex with age (Frith and Frith, 2003; Klindt et al., 2017; Poznyak et al., 2019), and socioeconomic status (SES) is a well-known general risk factor for psychopathology (Wadsworth and Achenbach, 2005) and it is also associated with mentalization (Mankus et al., 2016).

## Results

### Descriptive Statistics and Assumptions of the Models

Table 1 shows the descriptive statistics for the different measures of mentalization, symptoms, functioning and well-being, along with the correlations between them. All the significant correlations show the expected direction. All the regression models tested met the assumptions of independent errors (Durwin–Watson test), homoscedasticity (Plot of standardized predicted values against standardized residuals), normality of residuals (P–P plot), absence of multicollinearity (VIF and tolerance), and of influential cases (Cook’s distance).

**TABLE 1:** Correlations and descriptive statistics.

	**1**	**2**	**3**	**4**	**5**	**6**	**7**	**8**	**9**	**10**	**11**
1. Self	−										
2. Others	0.09	−									
3. S. Total	–0.12	–0.06	−								
4. S. Int	–0.12	–0.09	0.74**	−							
5. S. Ext	–0.10	0.01	0.88**	0.45**	−						
6. F. Gen	0.15*	0.26**	−0.50**	−0.23**	−0.41**	−					
7. F. Social	0.13	0.20**	−0.42**	−0.46**	−0.23**	0.50**	−				
8. F. Role	0.14*	0.24**	−0.48**	–0.13	−0.42**	0.91**	0.30**	−			
9. WB SE	0.30**	0.01	−0.30**	−0.34**	−0.22**	0.32**	0.25**	0.22**	−		
10. WB Hap	0.10	0.19**	−0.39**	−0.36**	−0.26**	0.62**	0.38**	0.59**	0.27**	−	
11. WB Tran	0.31**	0.16	–0.10	–0.07	–0.14	–0.02	0.08	–0.04	0.20*	0.03	−
Mean	73,89	14,57	1,35	1,29	0,94	25,71	32,16	28,49	21,50	4,51	34,82
SD	15,34	4,95	1,00	1,00	1,00	9,84	10,00	9,82	5,42	1,22	12,71

*p < 0.05 and **p < 0.01. S. Total; Symptoms Total; S. Int: Symptoms Internalizing; S. Ext: Symptoms Externalizing; F. Gen: Functioning General; F. Social: Functioning Social; F. Role: Functioning Role; WB SE: Well-being Self-esteem; WB Hap: Well-being Happiness; and WB Tran: Well-being Transcendence.

### Aim 1. Self and Other Mentalizing Polarities and Dimensions of Mental Health

Table 2 shows that mentalization referred to others’ mental states was associated with all measures of functioning (*B* = 0.475, *p* < 0.0005 for general functioning; *B* = 0.380, *p* = 0.005 for social functioning; and *B* = 0.364, *p* = 0.004 for role functioning), and self-mentalizing was associated with measures of psychological well-being (*B* = 0.076, *p* < 0.0005 for self-esteem; *B* = 0.209, *p* = 0.002 for transcendence). No mentalizing dimension was associated with symptoms. Happiness was not associated with self-mentalizing (*B* = 0.002, *p* = 0.728), but it was associated with the capacity to mentalize others (*B* = 0.053, *p* = 0.002).

**TABLE 2:** Association between mentalizing dimensions and different aspects of mental health (*n* = 214).

	**Self**		**Others**	
	***B* (*p*)**	**95% CI**	***B* (*p*)**	**95% CI**
*Symptoms*				
Total	-0.008 (0.056)	−0.017 to 0.0002	−0.003 (0.860)	−0.031 to 0.026
Internalizing	−0.006 (0.167)	−0.014 to 0.002	−0.025 (0.067)	−0.052 to 0.002
Externalizing	−0.008 (0.085)	−0.017 to 0.001	0.013 (0.394)	−0.016 to 0.041
*Functioning*				
General	0.051 (0.189)	−0.025 to 0.128	**0.475 (<0.0005)**	**0.228 to 0.721**
Social	0.040 (0.339)	−0.042 to 0.123	**0.380 (0.005)**	**0.113 to 0.646**
Role	0.058 (0.132)	−0.018 to 0.135	**0.364 (0.004)**	**0.119 to 0.609**
*Well-being*				
Self-esteem	**0.076 (<0.0005)**	**0.034 to 0.117**	0.117 (0.093)	−0.020 to 0.253
Happiness	0.002 (0.728)	−0.008 to 0.012	**0.053 (0.002)**	**0.020 to 0.087**
Transcendence	**0.209 (0.002)**	**0.079 to 0.340**	0.141 (0.534)	−0.307 to 0.589

*Results are adjusted for age, sex and socio-economic level in all cases. In case of functioning and well-being, results are additionally adjusted for total psychopathological symptoms. Significant B values are highlighted in bold.*

### Aim 2. Levels of Self and Other Mentalizing and Dimensions of Mental Health

Table 3 shows levels of self-mentalizing (attention vs. comprehension) and other-mentalizing (non-attachment based mentalization of “insignificant” or general others, vs. mentalizing of well-known attachment figures). Again, symptoms were not associated with any mentalizing process, though the subdimensions of attachment-based (others) and comprehension (self) demonstrated associations in the expected direction. Thus, deeper levels of mentalizing within each polarity showed associations with the predicted aspect of mental health. There were only two exceptions: (1) Attachment-based mentalizing was associated with general (*B* = 0.727, *p* = 0.014) and role functioning (*B* = 0.638, *p* = 0.027), but not with social functioning, and (2) comprehension of one’s own mental states revealed an association with self-esteem (*B* = 0.267, *p* < 0.0005) and transcendence (*B* = 0.335, *p* = 0.043), but not with happiness. In fact, happiness was not associated with any dimension of mentalization, whereas self-esteem was associated with all but one (i.e., in case of attachment-based others the result is suggestive but not significant: *B* = 0.299, *p* = 0.050).

**TABLE 3:** Association between levels of self- and others- mentalizing and different aspects of mental health (*n* = 214).

	**Self attention**		**Self comprehension**		**Others non-attachment based**		**Others attachment based**	
	***B* (*p*)**	**95% CI**	***B* (*p*)**	**95% CI**	***B* (*p*)**	**95% CI**	***B* (*p*)**	**95% CI**
*Symptoms*								
Total	0.005 (0.609)	−0.016 to 0.027	−0.017 (0.096)	−0.037 to 0.003	0.047 (0.347)	−0.051 to 0.144	−0.039 (0.239)	−0.105 to 0.026
Internalizing	0.005 (0.624)	−0.015 to 0.025	−0.015 (0.109)	−0.034 to 0.003	−0.051 (0.278)	−0.143 to 0.041	−0.012 (0.693)	−0.074 to 0.049
Externalizing	0.003 (0.775)	−0.018 to 0.025	−0.011 (0.286)	−0.031 to 0.009	0.095 (0.061)	−0.005 to 0.195	−0.045 (0.187)	−0.112 to 0.022
*Functioning*								
General	0.039 (0.677)	−0.145 to 0.223	0.094 (0.299)	−0.084 to 0.273	0.100 (0.816)	−0.749 to 0.949	**0.727 (0.014)**	**0.152 to 1.302**
Social	0.167 (0.107)	−0.036 to 0.369	0.020 (0.838)	−0.172 to 0.212	0.515 (0.282)	−0.426 to 1.456	0.264 (0.409)	−0.365 to 0.892
Role	0.022 (0.808)	−0.159 to 0.204	0.113 (0.208)	−0.063 to 0.289	−0.032 (0.940)	−0.871 to 0.806	**0.638 (0.027)**	**0.074 to 1.203**
*Well-being*								
Self-esteem	−**0.172 (0.001)**	−**0.268 to** −**0.075**	**0.267 (<0.005)**	**0.176 to 0.358**	−0.054 (0.812)	−0.498 to 0.391	0.299 (0.050)	0.000 to 0.598
Happiness	0.010 (0.409)	−0.014 to 0.035	−0.004 (0.760)	−0.027 to 0.020	0.057 (0.327)	−0.057 to 0.171	0.050 (0.198)	−0.026 to 0.126
Transcendence	0.173 (0.346)	−0.189 to 0.535	**0.335 (0.043)**	**0.011 to 0.658**	0.036 (0.965)	−1.590 to 1.663	0.234 (0.669)	−0.847 to 1.316

*Results are adjusted for age, sex and socio-economic level in all cases. Results are additionally adjusted for total psychopathological symptoms in case of functioning and well-being. Significant B values are highlighted in bold.*

## Discussion

### Self and Other Mentalization Polarities and Dimensions of Mental Health

Research suggests that the capacity of the mind to understand one’s own and others’ minds is a key factor for mental health (Luyten et al., 2020). Most studies have focused on the association between global measures of mentalizing and specific disorders. In contrast, very few studies have analyzed the association between specific mentalizing polarities and global measures of mental health. The first aim of this study was to analyze whether self and other polarities of mentalizing were associated with three widely accepted elements of global mental health, that is: symptoms, functioning and measures of psychological well-being (self-esteem, happiness, and motivation to life-goals) as an operationalization of what recent discussions define as “internal equilibrium” or internal functioning (Galderisi et al., 2015, 2017; World Health Organization (WHO), 2018).

In line with our predictions, the capacity to understand others’ mental states was associated with all measures of functioning, while the capacity to be aware of one’s own mental states was associated with self-esteem and transcendence (i.e., capacity to stablish life goals), but not with happiness.

The hypothesized association between happiness and self-mentalizing was possibly not supported because the current measure of well-being was based only on the teachers’ reports, that is, on the observation of adolescent behavior in the academic environment. In the case of well-being, self-reports are especially important. Future studies with more comprehensive measures might shed new light on this point. Interestingly, happiness is associated with mentalizing referred to others’ mental states. This result has two possible explanations: (1) the aforementioned reason, whereby the measure of happiness is based on teachers’ observation in the academic context where happiness is primarily experienced through social interaction; and (2) the importance of the social network for emotional well-being (Chu et al., 2010). Most studies support that social relationships and the support they provide are key factors for happiness (Cohen and Wills, 1985; Baumeister and Leary, 1995; Garcia-Carrion et al., 2019). Thus, if good mentalizing of others provides better social relationships, then it becomes an “indirect” factor for emotional well-being.

Further, the role of happiness in mental health has been recently questioned (Galderisi et al., 2015), which is an important consideration in light of our negative result. While the notion of mental health has evolved across one century to reach beyond symptoms (Bertolote, 2008), the initial WHO definition of mental health including well-being (World Health Organization (WHO), 2001) is purported to be excessively organized around an hedonic and eudaimonic perspective (Galderisi et al., 2017). Thus, it is possible that no association between self-mentalizing and happiness was revealed because happiness, as a hedonic state, is not a consistent dimension of the “*dynamic internal equilibrium”* referred by Galderisi and colleagues as the foundation of mental health. Future studies with more complete measures of emotional well-being should clarify this result, along with studies addressing the broader notion of “*dynamic internal equilibrium*,” represented here by the other two measures of psychological well-being, self-esteem and transcendence, along with happiness.

### The Lack of Association Between Mentalization Polarities and Internalizing or Externalizing Symptoms

Regarding symptoms, it was predicted that self-mentalizing should be associated with internalizing symptoms, while externalizing problems might be associated with self- and also other-mentalizing. Results did not support any association between polarities and symptoms. This is an unexpected finding for three reasons. Firstly, literature suggests that mentalizing is a resilience factor because it lacks when mental health lacks (for a review, see Sharp et al., 2008; Katznelson, 2014), and it is a common ingredient in all psychological treatments, that is: to restore mental health, mentalization must be improved (Allen et al., 2008). This suggests an association between mentalizing and mental health which should be applicable to all the three evaluated elements: functioning, well-being, and also symptoms. Secondly, previous studies utilizing global measures of mentalizing found no association with general psychopathology (e.g., Ballespí et al., 2018), but it was expected that investigation with a multidimensional measure of mentalizing would provide different results. Finally, albeit limited, there is evidence supporting the association between the role of mentalizing in the development of internalizing and externalizing problems (Sharp et al., 2008). Nonetheless, this evidence approaches the association from either a specific psychopathological condition (e.g., autism, sexual abuse, complex trauma, and borderline symptoms; e.g., Ensink et al., 2016) or a *trans*-generational point of view (i.e., maternal reflecting function moderating children externalizing difficulties; e.g., Ensink et al., 2017; Dejko–Wańczyk et al., 2020), or comprise a longitudinal design to analyze the trajectories of externalizing problems based on mentalizing variations (Morosan et al., 2020).

Our study design was cross-sectional and focused on a simple intra-individual association: to what extent one’s own mentalizing capacities are associated to one’s own symptoms. Current findings do not support the relationship between mentalizing polarities and symptoms in spite of using measures of higher order factors of psychopathology (internalizing, externalizing, and total; Caspi et al., 2014). Several possibilities exist for this lack of association. Firstly, the multi-informed measure of symptoms is based on parent- and teacher-reports, but it lacks adolescents’ self-evaluation. This is a limitation of this study. However, parent and teacher assessments of functioning are indeed associated with mentalizing. As such, the lack of association between symptoms and mentalization stands as an unresolved question. Secondly, it is possible that more specificity is required not only in the multidimensional assessment of mentalizing, but also regarding psychopathology. Perhaps the imbalance in mentalizing polarities is specific for different psychopathological conditions (Luyten et al., 2020) but it fades away when symptoms are grouped in higher-order factors (internalizing, externalizing, and “*p*” factor). This new possibility deserves attention.

The aim of this study was not to analyze specific disorders but general symptoms in a non-clinical sample. However, the lack of association between mentalizing polarities and higher-order factors of symptoms lead us to explore the clinical dimensions of the ASEBA system. More specificity in this regard did not change results. That is, when using the specific clinical scales (e.g., anxious-depressed, anxious-withdrawn) instead of the general factors (internalizing, externalizing, and general problems) no association between mentalizing polarities and the eight clinical scales of ASEBA was revealed, and very few associations appeared with the six DSM-oriented scales. Specifically, anxiety problems showed association with self-attention and oppositional-defiant disorders showed association with others’ mentalizing. More specificity did not change results. Of course, there is some contradiction in checking for clinical dimensions in a non-clinical sample, even when using a dimensional approach (Achenbach, 2020a,b). However, it is possible that the association between mentalizing and symptoms is not dimensional or progressive but qualitatively different in depending on the level of psychopathology. This might explain why results are different in clinical samples, where mentalizing is always impaired. Thus, it is possible that the association with functioning but not with psychopathology in the current study is due to the nature of the sample, where variability regarding functioning and well-being (usually high in non-clinical populations) is possibly different than variability of symptoms (usually low), even when using higher-order factors (internalizing, externalizing, and global). Studies ranging all the span of severity (from non-clinical to clinical levels) might reveal different results.

### Association Between Levels of Self- and Other- Mentalizing and Level of Mental Health

In contrast to symptoms, the levels of self- and other-mentalization fit predictions regarding functioning and well-being with two exceptions. Regarding functioning, while attachment-based mentalizing was found to be associated with general and role functioning, it was not associated with social functioning. This is particularly intriguing because the general measure of other-mentalizing (Table 2) was indeed associated with social functioning. Given that social functioning involves interaction with various individuals, including attachment figures and insignificant others, there is an association with “global” other-mentalizing but not with “partial” specific measures.

This leads us to question what types of measures of other-mentalizing should be used to best evaluate this ability. There are insignificant variations in standardized image-based measures such as the Movie for the Assessment of Social Cognition (MASC; Dziobek et al., 2006) or the Reading the Mind in the Eyes Test (RMET; Baron-Cohen et al., 2001) usually measuring mentalization based on external cues, compared to measures based on attachment-interviews (e.g., the Reflective Function Scale; Luyten et al., 2019), which are based on internal cues. It is possible that these variations have consequential implications in the multidimensional analysis of mentalizing and mental health. Knowing that assessment methods differ in the depth of mentalizing they elicit, and that therefore results may vary according to the assessment procedure, the election of the procedure should be made with caution and the comparison among studies should take variation in measure types into account.

With regard to well-being, comprehension of one’s own mental states was positively associated with self-esteem and to transcendent life goals, but not with happiness. In fact, as in the case of social functioning, the association of happiness with the global measure of other-mentalizing (Table 2) disappears when analyzing the sub-scores of the factor (i.e., attachment-based or referred to well-known others vs. non-attachment based or referred to general others; Table 3). Again, this suggests that an association only exists when combining both aspects of other-mentalizing (i.e., the general social knowledge used to solve the picture-based assessment, along with the capacity to read the specific mental states of particular close-others). Since the measure of well-being comes from teacher reports in the ASEBA system, these results should be interpreted with caution. Finally, since the role of hedonic well-being as a dimension of mental health has been questioned (Galderisi et al., 2017), our different results with happiness than with the other measures of internal well-being (i.e., self-esteem and transcendence) could be coherent with this perspective.

Interestingly, self-esteem was found to be positively associated with comprehension and negatively associated with attention to one’s own emotions. This is consistent with previous findings and supports the idea that comprehension is more vital to self-function than simple attention. Too much attention to emotions, particularly if it lacks understanding of said emotions, can be overwhelming and it is usually associated with emotional dysregulation, which blocks mentalizing (Fonagy et al., 2005). In contrast, comprehension of emotional states is a coping skill to deal with emotional pain (Allen et al., 2008) and also leads to self-knowledge (Fonagy and Target, 1997), which is path to self-acceptance (Wilson, 2009). Therefore, it is reasonable that self-esteem is associated with good understanding of one’s self.

### Limitations and Recommendations for Further Research

This is a first study showing a specific association between self and other polarities of mentalizing and aspects of self-other healthy functioning. Above all, the findings reported in the present study should be replicated before drawing significant interpretations. Further, limitations to the research do exist. First, the cross-sectional design impedes the possibility of establishing causal relationships. As such, it is unknown whether the imbalances in self and other polarities of mentalizing are the cause or the consequence of the mental health dimensions that were analyzed. Moreover, although a multi-informant-based method was used for most variables, adolescent self-report was lacking for most measures. Future studies including self-reports may further highlight whether no association between mentalization polarities and symptoms in general population is indeed present. Finally, better measures of mentalization polarities are required. The *zeitgeist* of the study of this higher order cognition has just begun, and the problem of how measuring such a complex function still lacks a solution, especially regarding the self- polarity. In this sense, the TMMS-24 is a widely used and psychometrically well-stablished measure of emotional self-awareness, but it is a self-report, and there is currently no method by which researchers or clinicians may assess subprocesses or levels of self-mentalizing, with the only exception of the LEAS (i.e., the *Levels of Emotional Awareness Scale*; Lane et al., 1990), which was dismissed for this study because it is longer (20 social situations about which the adolescent should write with no limit of words) and not adapted to Spanish and Catalan population. In this sense, the importance of refining the multidimensional assessment of mentalizing has been already highlighted (Luyten et al., 2020).

While some limitations of this research are present and it would benefit from replication, this study also benefits from prominent strengths. To our knowledge, this research is novel in nature–no studies to date have analyzed the association between self- and other- mentalization polarities and a multidimensional measure of mental health in a non-clinical population. This multidimensional approach inherently considers recent developments in the approach to mental health, which is *trans*-diagnostic and *trans*-symptomatic [Galderisi et al., 2017; World Health Organization (WHO) (2018)] involving social and role functioning and measures of “internal equilibrium” along with different types of symptoms. Further, both symptoms and functioning indicators come from multi-informed measures based on Achenbach’s System for Empirical-Based Assessment (Achenbach, 2020a), which provides a dimensional perspective consistent with modern approaches to mental health (Cuthbert, 2014).

## Conclusion and Implications for Mental Health

To our knowledge, this is the first study to show a specific association between self- and other- mentalizing polarities and different aspects of mental health. Despite the fact that no mentalizing dimension was associated with symptoms, this is the first study to date to show an association between self-other mentalizing polarities and aspects of self-other function. This is not only suggestive of adjusting interventions to the specific aspects of mentalization dimensions that require treatment, but it also highlights the importance of mentalizing for salutogenesis, even in a non-clinical sample. In this sense, this novel research supporting the association between mentalization and mental health, even in a sample from non-clinical population, stresses the importance of fostering mentalization not only in the clinical contexts, when mental health suffers because a disorder was developed, but also and very especially when the disorder still lacks and in order to prevent its development.

Mentalization-based treatments help to restore mental health when it is lacking, and this means to work on mentalization abilities under the impairing effect of a disorder (and thus, in suboptimal conditions). Why do not foster such important abilities when a disorder is not present? According to Heckman’s equation (Heckman, 2020b), any investment in human potential prior to the onset of a clinical condition might provide a higher return the earlier is done, always higher than investing the same efforts under impairing conditions. This is suggestive of nurturing mentalization not only in clinical but also in general population, where mentalization is also associated to positive mental health, according to current results. However, this begs the question of why our societies and educational systems invest such financial resources and attention to traditional literacy (reading, writing, and reasoning) while invest a relative absence of resources in the “emotional literacy” of the general population. The school formal curriculum is aimed at training children in competences of unquestionable relevance (i.e., languages, mathematics, social and natural knowledge, sciences, physical education, arts, and digital learning) across a minimum of 10 years. Out of school activities are aimed at providing specialized attention to improving sports, playing a musical instrument, speaking a new language or training other skills. Interestingly, we only seek specialized attention for our socio-emotional competences once there is a clinically diagnosable issue. The present results support a call to invert this situation.

Current findings support an association between mentalization polarities and mental health in a sample of adolescents from general population. This association highlights the importance of promoting mentalization in developmental non-clinical population, when mental health is not disrupted and learning conditions are optimal. Fostering mentalization across the developmental stage, in adolescence or possibly earlier, may not only meet the standards set forth by Heckman (Heckman, 2020a) and the idea to nurture a *“dynamic internal equilibrium”* which is the foundation of mental health (Galderisi et al., 2015). Moreover, fostering mentalization across development: (1) might help to develop mentalizing in a more natural manner than the intensive treatments do once clinical threshold is met, (2) might provide resilience throughout development as mentalizing is promoted from early stages, and (3) might help to produce an adult with better mentalizing abilities and therefore a generation more able to mentalize the subsequent one. Finally and most importantly, fostering mentalization across development may incorporate one of the UNESCO’s challenges (UNESCO, 2017, 2019) for the Education of the millennium citizen: this will educate individuals to properly regulate their emotions and to help others to do so.

## Data Availability Statement

The original contributions presented in the study are included in the article/Supplementary Material further inquiries can be directed to the corresponding author/s.

## Ethics Statement

The studies involving human participants were reviewed and approved by Ethics Committee of the Universitat Autònoma de Barcelona (CEEAH, Spain; CEEAH no. 2603). Written informed consent to participate in this study was provided by the participants and/or their legal guardian/next of kin.

## Author Contributions

SB conceptualized and investigated the study and wrote the manuscript. SB and JV contributed to data curation and visualization, defined the methodology, and performed formal analysis. All authors acquired funding and provided resources. CS, LC, and NB-V validated the data. All authors critically reviewed the manuscript.

## Conflict of Interest

The authors declare that the research was conducted in the absence of any commercial or financial relationships that could be construed as a potential conflict of interest.
